# Family-Based GWAS of Cognitive Endophenotypes Reveals Genetic Architecture of Memory and Executive Function in Alzheimer’s Disease

**DOI:** 10.3390/cimb48050442

**Published:** 2026-04-24

**Authors:** Kesheng Wang, Xueying Yang, Gayenell Magwood, Chun Xu, R. Osvaldo Navia, Jean Neils-Strunjas, Xiaoming Li

**Affiliations:** 1 Department of Biobehavioral Health & Nursing Science, College of Nursing, University of South Carolina, Columbia, SC 29208, USA; magwoodg@mailbox.sc.edu; 2 South Carolina SmartState Center for Healthcare Quality, Arnold School of Public Health, University of South Carolina, Columbia, SC 29208, USA; xueyyang@mailbox.sc.edu (X.Y.); xiaoming@mailbox.sc.edu (X.L.); 3 Department of Health Promotion, Education and Behavior, Arnold School of Public Health, University of South Carolina, Columbia, SC 29208, USA; 4 Department of Health and Biomedical Sciences, College of Health Affairs, University of Texas Rio Grande Valley, Brownsville, TX 78520, USA; chun.xu@utrgv.edu; 5 Division of Geriatrics and Palliative Medicine, John W. Deming Department of Medicine, Tulane University School of Medicine, New Orleans, LA 70112, USA; onavia@tulane.edu; 6 Department of Communication Sciences and Disorders, Arnold School of Public Health, University of South Carolina, Columbia, SC 29208, USA; neilsstj@mailbox.sc.edu

**Keywords:** Alzheimer’s disease, cognitive performance, genome-wide association study, family-based analysis, single nucleotide polymorphisms, memory, *TOMM40*, aging, pathway

## Abstract

Alzheimer’s disease (AD), the most common cause of dementia, is characterized by progressive memory and cognitive decline. Conventional genome-wide association studies (GWAS) comparing AD cases and controls may miss genetic influences that act along a continuum of cognitive function. Using data from 3007 participants in the National Institute on Aging Late-Onset Alzheimer’s Disease Family Study (NIA-LOAD GWAS), we conducted a family-based GWAS of eight quantitative cognitive phenotypes encompassing episodic memory (Logical Memory IA and IIA), working memory (Digit Span Forward, Backward, and Ordering), and semantic fluency (Animal, Fruit and Vegetable, and Vegetable Fluency). Family-based association testing in PLINK v1.9 identified numerous single nucleotide polymorphisms (SNPs) associated with cognitive phenotypes at genome-wide significant (*p* < 5 × 10^−8^) levels. Notably, genome-wide significant variants with cognatic functions were localized to genes implicated in synaptic function, neurodevelopment, and neurodegeneration, including *TOMM40* (rs2075650), *ERBB4* (rs1521543), *APLP2* (rs12281267, rs959354), *PTPRD* (rs1353983, rs970347, rs1392511), *NCAM2* (rs2826728), *GRM7* (rs6788201), *PAX5* (rs2988003, rs2381595), *NRG1* (rs16875655), and *NRG3* (rs1937957). Furthermore, the *TOMM40* (rs2075650) was significantly associated with AD as a binary outcome (*p* = 4.60 × 10^−24^) and *APLP2* (rs12281267, rs959354), *APOE* (rs405509), *PTPRD* (rs1353983, rs970347, rs1392511) were associated with AD (*p* < 0.001). Additionally, several pathways including the *ERBB4* signaling pathway (adjusted *p* = 2.82 × 10^−3^), driven by *ERBB4*, *NRG1*, and *NRG3* may contribute to cognitive impairments. This study provides a comprehensive resource of cognitive endophenotype associations in AD families, advancing understanding of the genetic architecture underlying memory, executive function, and cognitive aging, and highlights new therapeutic targets for replication and functional follow-up.

## 1. Introduction

Alzheimer’s disease (AD) is the most common cause of dementia globally, accounting for approximately 60–80% of all dementia cases [1]. AD is a chronic, progressive neurodegenerative disorder marked by declines in memory, language, executive function, and visuospatial skills [2]. At the neuropathological level, AD is characterized by the extracellular accumulation of amyloid-β (Aβ) plaques and the intracellular aggregation of neurofibrillary tangles composed of hyperphosphorylated tau protein, culminating in neuronal loss, synaptic dysfunction, and widespread brain atrophy [3,4]. The burden of AD is mounting: as of 2024, more than 55 million people worldwide live with dementia, and this number is projected to surge to 139 million by 2050 [5]. In the United States (U.S.) alone, AD affects more than 6.9 million persons aged 65 and older, exacting tremendous social, emotional, and economic costs on patients, families, and healthcare systems [1]. Despite extensive scientific effort, effective disease-modifying therapies remain elusive, underscoring the critical need to deepen our understanding of the genetic and biological mechanisms driving AD risk and progression [4].

AD is a complex multifactorial disorder influenced by both genetic and environmental factors. The majority of cases are late-onset AD (LOAD; onset after age 65) [6], which is highly heritable (60–80%) and polygenic in nature [7]. While rare early-onset forms are driven by highly penetrant mutations in the APP, PSEN1, and PSEN2 genes [8,9], the ε4 allele of APOE gee remains the strongest genetic risk factor for LOAD [10,11]. In addition, genome-wide association studies (GWAS) have identified numerous susceptibility loci, including CLU, PICALM, BIN1, ABCA7, and TREM2, implicating pathways related to amyloid processing, lipid metabolism, immune regulation, and endosomal function [12,13,14,15,16]. Despite these advances, the translation of genetic discoveries into improved early detection and therapeutic strategies remains limited.

Compared to studies of AD risk, relatively less attention has been paid to the genetic architecture of cognitive function, which may precede clinical disease onset. Both general and domain-specific cognitive performance (e.g., memory, executive function, and language) is moderately to highly heritable and influenced by polygenic factors [17,18]. Increasing evidence suggests that cognitive phenotypes may serve as intermediate endophenotypes linking genetic variation to AD risk [19,20]. For example, variants in APOE and TMEM106B have been linked to both memory performance and risk for AD and other neurodegenerative disorders [21,22]. Large GWAS of general cognitive function or intelligence in population-based cohorts have demonstrated genetic overlap with health outcomes, neurodegenerative disorders, and psychiatric conditions [17,18,23]. Furthermore, methodological advances in cognitive–genetic research increasingly emphasize interaction modeling (e.g., SNP × age), pleiotropy across cognitive domains, and the use of continuous quantitative endophenotypes rather than dichotomous outcomes. For example, transcription-centric SNP–age interaction models in large human tissue datasets demonstrate that genetic effects can vary substantially across the lifespan [24]. Similarly, polygenic indices derived from GWAS show age-dependent associations with cognitive performance, suggesting dynamic genetic influences over time [25]. Despite these developments, many studies still rely on case–control disease status rather than detailed cognitive phenotypes [26]. Moreover, numerous GWAS focus on global cognitive ability using brief, cross-sectional assessments, rather than comprehensive neuropsychological batteries capturing domain-specific functions such as episodic memory, working memory (e.g., digit span and sequencing), and semantic fluency [19]. These finer-grained measures may be more sensitive to early decline relevant to AD. However, the domain-specific effects of genetic variants—including established risk loci (e.g., *TOMM40*/*APOE*) and emerging candidates (e.g., *PTPRD*, *NCAM2*, *GRM7*, *NRG3*)—remain poorly understood, particularly in preclinical stages. Family-based GWAS offer a robust framework to address this gap by mitigating population stratification and enhancing detection of modest, heritable effects [26,27].

Despite the identification of numerous AD susceptibility loci, several critical gaps remain. First, the genetic architecture of domain-specific cognitive functions—particularly those assessed via detailed neuropsychological tests—remains poorly characterized, and even less so within family-based designs [28]. Second, the majority of GWAS have focused on clinically diagnosed AD rather than continuous quantitative cognitive traits, which may serve as earlier and more sensitive markers of disease risk [29]. Third, the biological mechanisms linking genetic variants to cognitive performance are still incompletely understood, limiting the translation of genetic discoveries into therapeutic targets [30,31]. To address these gaps, we analyzed data from 3007 participants in the National Institute on Aging Late-Onset Alzheimer’s Disease (NIA-LOAD) Family Study. Using a family-based GWAS approach (QFAM in PLINK v1.9), we investigated eight quantitative cognitive phenotypes spanning episodic memory, working memory, and semantic fluency. Our aims were to identify genetic variants associated with domain-specific cognitive performance, characterize genome-wide significant and suggestive genes/loci, and provide a resource for future replication and functional studies.

## 2. Materials and Methods

### 2.1. Study Population

Data were derived from the NIA-LOAD Family Study: Genome-Wide Association Study for Susceptibility Loci (dbGaP accession: phs000168.v2.p2). The NIA-LOAD study is a multicenter, family-based cohort designed to identify genetic factors influencing the risk and progression of LOAD [32]. In total, the dataset includes 3007 participants (including 1266 with AD) from 1235 multiplex families, encompassing both affected and unaffected relatives. All participants underwent structured clinical evaluation, neurological examination, and neuropsychological testing using standardized protocols across study sites. Diagnosis of AD was made through consensus review incorporating cognitive testing, medical history, and informant interviews. Additional demographic variables (age, sex, education, and family structure) were recorded and included as covariates in downstream analyses.

### 2.2. Cognitive Function Measures

Eight neuropsychological measures representing episodic memory, working memory, and verbal fluency domains were analyzed as quantitative traits. Higher scores indicate better cognitive performance.

1. Logical Memory IA (Immediate Recall)—number of story elements recalled immediately after presentation (episodic memory).
2. Logical Memory IIA (Delayed Recall)—number of story elements recalled after a delay (episodic memory retention).
3. Digit Span Forward—ability to repeat digits in the same order (short-term memory and attention).
4. Digit Span Backward—ability to recall digits in reverse order (working memory).
5. Digit Ordering—ordering of digits by magnitude (sequencing and executive control).
6. Animal Fluency—number of animal names produced within one minute (semantic subcategory fluency).
7. Fruit and Vegetable Fluency—number of fruits and vegetables produced in one minute (semantic category fluency).
8. Vegetable Fluency—number of vegetables produced in one minute (semantic subcategory fluency).

Reliability estimates for commonly used quantitative cognitive phenotypes are well documented in the neuropsychological literature, typically reported as test–retest reliability or internal consistency [33,34]. Episodic memory measures such as Logical Memory IA (immediate recall) and Logical Memory IIA (delayed recall) generally demonstrate good reliability, with coefficients ranging from approximately 0.70 to 0.90 and delayed recall often showing slightly higher stability [35,36]. Working memory measures from the Digit Span task also exhibit moderate to high reliability, with Digit Span Forward ranging from 0.70 to 0.85, Digit Span Backward from 0.75 to 0.85, and Digit Span Ordering (Sequencing) from 0.80 to 0.90 [37,38]. Semantic fluency tasks, including Animal Fluency and category-based fluency tasks (e.g., fruits and vegetables), typically show good reliability (approximately 0.75–0.90), although narrower categories such as vegetable fluency alone may yield slightly lower estimates due to restricted variability [35,39]. These measures are widely considered sufficiently reliable for use as quantitative phenotypes in cognitive aging and AD research [33,34,40].

### 2.3. Descriptive Statistics

Categorical variables were presented as frequencies and percentages, while continuous variables were presented as range, mean and standard deviation (SD). The chi-square test was used to compare the frequency of AD across gender and racial groups. An independent *t*-test was used to compare the means in continuous variables between the AD and non-AD groups. Spearman correlation was used to examine the relationships among age, education, and cognitive measures.

### 2.4. Genotyping and Quality Control

DNA was extracted from peripheral blood samples and genotyped using the Illumina Human610-Quad BeadChip (Illumina Inc., San Diego, CA, USA), which assays ~620,000 SNPs genome-wide. Quality control (QC) procedures followed standard GWAS pipelines. SNPs were excluded if they had a call rate < 95%, minor allele frequency (MAF) < 1%, or Hardy–Weinberg equilibrium (HWE) *p* < 1 × 10^−6^ in founders. Individuals were removed if they had genotype call rates < 95%. Genomic positions were aligned to the NCBI36 (hg18) human reference genome. Imputation was not applied, as the original NIA-LOAD release provides directly genotyped high-density markers and family-based imputation across pedigrees is complex.

### 2.5. Genome-Wide Association Analyses

#### 2.5.1. Family-Based Genome-Wide Association Analyses of Quantitative Phenotypes

Family-based genome-wide association analyses of cognitive measures were conducted using PLINK v1.9 [41,42]. For continuous cognitive measures, we used the QFAM procedure (plink–qfam), which decomposes genotype and phenotype values into between-family and within-family components. This design preserves power while accounting for family structure and relatedness and applies a permutation-based test to control type I error. The qfam-total options were used to combine the within- and between-family components (i.e., total association) for quantitative traits, while mperm 10000 was used to specify 10,000 permutations (max-T style) for empirical *p*-values for each phenotype. Genome-wide significance was defined as *p* < 5 × 10^−8^ and suggestive significance as *p* < 1 × 10^−5^ [43]. Manhattan plots were generated to visualize the genome-wide distribution of association *p*-values and assess potential test statistic inflation.

To account for multiple testing across the eight correlated cognitive phenotypes, we estimated the effective number of independent traits (Meff) based on their correlation structure using eigenvalue decomposition of the phenotype correlation matrix. We applied the method of Li and Ji (2005) [44], which is widely used in GWAS and multi-phenotype analyses because it provides a computationally efficient approximation of the number of independent tests while accounting for trait correlations. Based on the correlation structure of the eight phenotypes (after adjustment for covariates), the effective number of independent traits was estimated to be Meff = 4.83. Accordingly, we applied a phenotype-level correction to the conventional genome-wide significance threshold, resulting in an adjusted threshold of *p* < 1.04 × 10^−8^ (i.e., 5 × 10^−8^/4.83).

To evaluate the statistical power of our study, we performed power calculations for genome-wide association testing of quantitative traits under an additive genetic model. Analyses assumed genotype coding of 0, 1, and 2 minor alleles, standardized phenotypes, and two-sided testing. Power was evaluated using the noncentral F distribution, with the noncentrality parameter defined by the proportion of variance explained (*R*^2^) by a given SNP, where $R^{2}\approx 2p(1-p)\beta^{2}$, with p  representing the minor allele frequency (MAF) and β the per-allele effect size. We considered a genome-wide significance threshold of $5\times 10^{-8}$, as well as a phenotype-adjusted threshold accounting for multiple correlated traits using the effective number of independent phenotypes (Meff = 4.83), yielding an adjusted threshold of $1.04\times 10^{-8}$. Power and minimum detectable effect sizes were calculated for each phenotype-specific sample size assuming a representative MAF of 0.20.

For each phenotype, we estimated (i) the statistical power to detect a modest effect size (β = 0.15) and (ii) the minimum detectable effect size (β and corresponding R^2^) required to achieve 80% power at both significance thresholds. Power calculations demonstrated that the study was underpowered to detect small effect sizes at genome-wide significance across all phenotypes. For an assumed effect size of β = 0.15 and MAF = 0.20, statistical power ranged from 1.1% for the largest phenotype (N = 1400) to <0.01% for the smallest phenotype (N = 460), indicating that such modest effects are unlikely to be detected reliably in the present sample. In contrast, the minimum detectable effect size required to achieve 80% power varied substantially by sample size. For phenotypes with N ≈ 1400, variants explaining approximately 2.8–3.0% of trait variance (β ≈ 0.295–0.308) were detectable. For intermediate sample sizes (N ≈ 900–1000), detectable effects increased to *R*^2^ ≈ 3.9–4.7% (β ≈ 0.35–0.38), while for the smallest phenotype (N = 460), only variants with large effects (*R*^2^ ≈ 8.2–8.9%; β ≈ 0.51–0.53) were detectable at 80% power. Detailed power estimates for each phenotype are provided in Supplementary Table S1.

#### 2.5.2. Genome-Wide Association Analysis of AD as a Binary Outcome

For comparison with disease status, genome-wide association analysis of AD was performed using logistic regression in PLINK v1.9. The model included the following covariates: sex, principal components (PC1–PC4) derived from genome-wide genotype data to control for population stratification, ethnicity (Hispanic vs. non-Hispanic as a categorical variable), and education (years), as well as cluster/family strata using within-cluster indicators to account for familial clustering. The first four PCs (PC1–PC4), which capture the major axes of genetic variation that correspond to ancestral differences (e.g., European, African, Asian, and admixed populations), were used as covariates to adjust for potential confounding due to population stratification.

### 2.6. Annotation and Functional Follow-Up

First, the sequences containing the associated SNPs were examined for microRNA binding sites, splicing sites, regulatory gene regions, and species-conserved regions using NIH-SNP Function Prediction (https://snpinfo.niehs.nih.gov/snpinfo/snpfunc.html (accessed on 18 December 2025)). Then, expression quantitative trait loci (eQTL) effects were explored using GTEx (v8) [45] and gtexr R package 0.2.1 [46].

### 2.7. Bioinformatic Analysis

The R package “ClusterProfiler” v.4.18.4 [47] was used to perform Gene Ontology (GO) enrichment (http://www.geneontology.org) [48] and Kyoto Encyclopedia of Genes and Genomes (KEGG) pathway (http://www.genome.jp/kegg/pathway.html (accessed on 13 January 2026)) [49] analyses of genes to evaluate whether the targeted genes were involved in important biological processes. Results were visualized by the R package “ggplot2” package [50] in R 4.4.3 (R Core Team, Vienna, Austria).

We performed GO enrichment with clusterProfiler: enrichGO (keyType = “ENTREZID”, pAdjustMethod = “BH”, pvalueCutoff = 0.05, readable = TRUE). For pathway enrichment we attempted clusterProfiler: enrichKEGG (organism = “hsa”) but, when KEGG access was unavailable, we used two fallback approaches: (a) ReactomePA: enrichPathway (organism = “human”) and (b) MSigDB KEGG-style gene sets via msigdbr + clusterProfiler::enricher (TERM2GENE). All enrichment analyses used Benjamini–Hochberg multiple-testing correction and significance threshold of adjusted *p* < 0.05 unless otherwise stated.

## 3. Results

### 3.1. Sample Characteristics

The NIA-LOAD dataset included 3007 individuals from 1235 multiplex families with complete genotype and neuropsychological data. There was no significant difference in the proportion of AD cases between males and females (Table 1). Similarly, age did not differ significantly between individuals with and without AD. The prevalence of AD was higher among White participants (53.3%) compared to African American participants (32.5%). In addition, individuals with AD had lower educational attainment (mean 12.8 years) compared to those without AD (mean 14.7 years).

Descriptive statistics for the eight cognitive measures are summarized in Table 2. Individuals with AD exhibited lower performance across all cognitive measures compared to those without AD. Spearman correlation analyses demonstrated that the eight cognitive measures were positively correlated with one another, reflecting shared cognitive domains. In addition, cognitive performance was positively associated with years of education and negatively associated with age (Supplementary Table S2).

### 3.2. Genome-Wide Association Study

**Figure 1:**
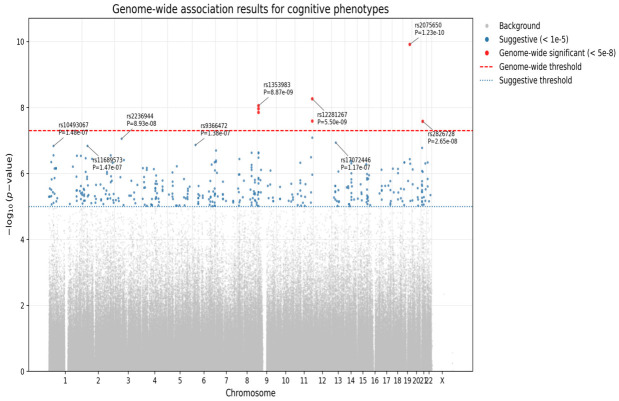
Manhattan plot based on GWAS of delayed recall (Logical Memory IIA). Each point represents a SNP plotted by chromosomal position and −log10(*p*-value). The red dashed line indicates genome-wide significance (*p* = 5 × 10^−8^), and the blue dotted line indicates suggestive significance (*p* = 1 × 10^−5^). Only independent lead SNPs (identified by LD clumping) are annotated. The strongest signal is observed at rs2075650 in the APOE/TOMM40 region on chromosome 19.

**Figure 2:**
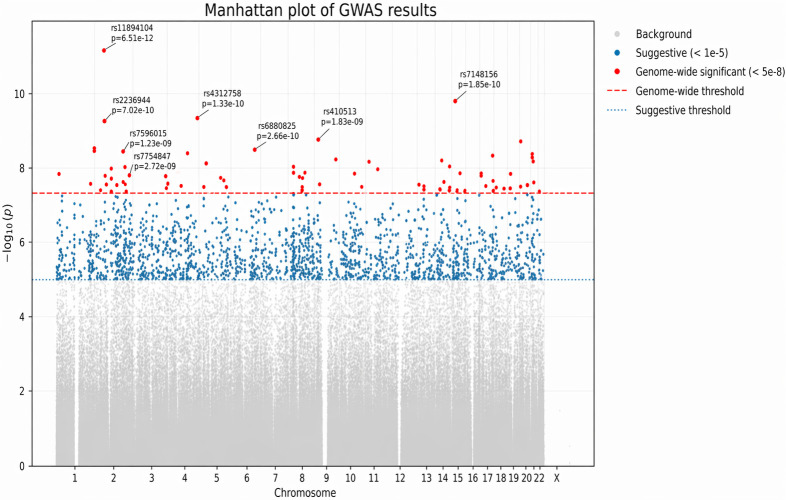
Manhattan plot based on GWAS of Digit Span Backward. Each point represents a SNP plotted by chromosomal position and −log10(*p*-value). The red dashed line indicates genome-wide significance (*p* = 5 × 10^−8^), and the blue dotted line indicates suggestive significance (*p* = 1 × 10^−5^). SNPs exceeding genome-wide significance are highlighted in red, while suggestive associations are shown in blue. Only independent lead SNPs (selected based on genomic distance) are annotated.

Family-based GWAS using PLINK’s QFAM procedure identified several or more loci for each cognitive measure with significant or suggestive associations (*p* < 5 × 10^−8^ and *p* < 1 × 10^−5^, respectively). A total of seven SNPs reached genome-wide significance for Logical Memory IIA (Delayed Recall) (Figure 1 and Table 3). Figure 1 shows that significant association signals were observed across multiple chromosomes, with the most prominent peaks located on chromosomes 9, 11, 19, and 21. Notably, the strongest signal was observed at rs2075650 on chromosome 19 within the TOMM40/APOE region. Additional genome-wide significant loci were identified in regions harboring genes previously implicated in neurodevelopment and synaptic function, including *PTPRD*, *NCAM2*, and *GRM7*. While several loci exceeded the conventional genome-wide significance threshold, others showed suggestive associations (*p* < 1 × 10^−6^), indicating potential additional signals requiring replication. Furthermore, 90 SNPs were significantly associated with Digit Span Backward (Figure 2 and Table 4). Figure 2 reveals that genome-wide association analysis identified multiple loci reaching genome-wide significance (*p* < 5 × 10^−8^), distributed across several chromosomes. The most significant association was observed at rs11894104 within *HSPC159||AFTPH* on chromosome 2 (*p* = 6.51 × 10^−12^), representing the strongest signal in the dataset. Additional genome-wide significant loci were detected on chromosomes 3, 4, 5, 8, 14, and 21, indicating a polygenic architecture underlying the cognitive phenotype. In addition, significant loci were observed for Digit Span Forward (20 SNPs), Digit Ordering (six SNPs), Logical Memory IA (one SNP), and Animal Fluency (one SNP). No SNP reached genome-wide significance for Fruit and Vegetable Fluency or Vegetable Fluency, although several suggestive signals were noted (*p* < 1 × 10^−5^). A total of 116 unique SNPs (*p* < 5 × 10^−8^) significantly associated with these cognitive functions are listed in Supplementary Table S3.

The top genome-wide significant SNPs for delayed recalls (Logical Memory IIA), immediate recall (Logical Memory IA) and Animal Fluency are presented in Table 3. The corresponding *p*-values for AD, Digit Span Backward, Digit Span Forward, and Digit Ordering are also presented in Table 3. Table 3 presents the chromosome number (Chr.), SNP rs number, and physical position. It also includes the SNP functional annotation (nearest gene), minor allele, MAF, and the corresponding *p*-values for AD and six cognitive measures.

The top genome-wide significant SNPs for Digit Span Backward (top seven SNPs), Digit Span Forward (top seven SNPs), and Digit Ordering (six SNPs) are presented in Table 4. The corresponding *p*-values for AD, delayed recalls (Logical Memory IIA), immediate recall (Logical Memory IA) and Animal Fluency are also presented in Table 4.

#### 3.2.1. Genome-Wide Significant SNPs for Episodic Memory (Logical Memory IA & IIA)

For Logical Memory IA (Immediate Recall), the leading signal was rs2075650 in *TOMM40* (*p* = 3.2 × 10^−8^), located within the APOE genomic region on chromosome 19 (Table 3). For Logical Memory IIA (Delayed Recall), seven SNPs reached genome-wide significance, including rs2075650 (*TOMM40*), rs12281267 and rs959354 (*APLP2*), rs1353983, rs970347, and rs1392511 (*C9orf123||PTPRD*), and rs2826728 (*NCAM2*) (all *p* < 5 × 10^−8^). Table 5 presents five flanking SNPs located within *TOMM40* and/or *APOE*. In addition to rs2075650, three SNPs (rs157580, rs8106922, and rs405509) demonstrated strong associations with AD, with *p*-values of 8.89 × 10^−8^, 6.49 × 10^−8^, and 3.70 × 10^−7^, respectively, while rs157580 was also associated with Logical Memory IIA (Delayed Recall, (*p* = 3.2 × 10^−7^)) and Logical Memory IA (Immediate Recall, (*p* = 3.2 × 10^−5^).

#### 3.2.2. Genome-Wide Significant SNPs for Working Memory and Attention (Digit Span Forward, Backward, and Digit Ordering)

For Digit Span Forward, the top seven SNPs included rs10759013 (*PTPRD*), rs1110399 (*SLC29A3*), rs11160011 (*SMEK1||PP8961*), rs3862188 (*OR13G1||OR6F1*), rs4312758 (*KIAA0232*), rs6788201 (*GRM7*), and rs880143 (*OR13G1||OR6F1*) (all *p* < 5 × 10^−8^) (Table 4). For Digit Span Backward, 90 genome-wide significant loci were identified, with the top seven signals being rs11894104 (*HSPC159||AFTPH*), rs2236944 (*GNAI2*), rs410513 (*LOC100129568||LOC137012*), rs4312758 (*KIAA0232*), rs6880825 (*YTHDC2||KCNN2*), rs7148156 (*GOLGA5*), and rs7571817 (*DNER*). For Digit Ordering, six genome-wide significant loci were detected: rs16971186 (*VPS18*), rs6714929 (*ICOS||LOC100132132*), rs4878454 (*MOBKL2B*), rs2988003 (*PAX5*), rs2381595 (*PAX5*), and rs9617659 (*PEX26||TUBA8*).

#### 3.2.3. Genome-Wide Significant SNPs for Semantic Fluency (Animal, Fruit, and Vegetable Fluency)

A single SNP, rs1937957 (*NRG3*), reached genome-wide significance for Animal Fluency (*p* = 4.7 × 10^−8^) (Table 3). Although no SNPs achieved genome-wide significance for Fruit and Vegetable Fluency or Vegetable Fluency, suggestive associations near *ZNF804A*, *CNTNAP2*, and *SYT1* genes implicated in language and semantic networks were observed (*p* < 1 × 10^−5^) (Supplementary Table S3).

#### 3.2.4. Cross-Phenotype Overlapped Genome-Wide Significant SNPs

Comparative analyses revealed substantial overlap among loci associated with different cognitive domains. For example, *TOMM40* variant (rs2075650) on chromosome 19 influenced both episodic memory and semantic fluency (Table 3), as well as AD with *p* = 4.60 × 10^−24^ (Table 3). Furthermore, *PTPRD* (several SNPs) on chromosome 9 and *NCAM2* on chromosome 21 emerged as a recurrent locus across Episodic Memory, Digit Span Forward, and Digit Ordering, suggesting pleiotropic effects on both memory and executive control (Table 3 and Table 4). *NRG3* on chromosome 10 is significantly associated with Anima Fluence and suggestively with Digit Ordering. *KIAA0232* (rs4312758) on chromosome 4 and *GNAI2* (rs2236944) on chromosome 3 were associated Episodic Memory, Working Memory and Animal Fluency (Table 4).

### 3.3. Functional Annotation and eQTL Analyses

We evaluated whether the 116 genome-wide significant variants were located in genomic regions with potential functional relevance using the NIH SNP Function Prediction tool (Supplementary Table S3). Several variants were found to reside in regulatory elements that may influence gene epression at transcriptional and post-transcriptional levels, including RNA splicing and stability. For example, rs7225748, located within *OR1P1P* and associated with Digit Span Backward, falls within a transcription factor binding site (TFBS). Variants in TFBSs can alter the affinity of transcription factor–DNA interactions, potentially creating, disrupting, or modifying binding motifs and thereby affecting transcriptional regulation and downstream phenotypic variation. Additionally, rs2291029 within *NUP85*, associated with both Digit Span Forward and Backward, is located in exonic splicing enhancers (ESEs) and exonic splicing silencers (ESSs)—cis-acting regulatory elements that modulate alternative splicing through interactions with splicing factors. Disruption of these elements may lead to altered transcript isoforms and functional consequences. Other variants, including rs2236944 within *GNAI2* and rs2075650 within *TOMM40*, were also predicted to have regulatory potential, further supporting a functional role for these loci. Collectively, these findings suggest that a subset of the identified variants may influence cognitive-related phenotypes through regulatory mechanisms affecting gene expression or RNA splicing.

Functional annotation using GTEx (v8) were performed for SNPs in Table 3 and Table 4. From the 27 SNPs, nine SNPs were found to have eQTL effects (Supplementary Table S4). rs2075650 (*TOMM40*) and rs2826728 (*NCAM2*) demonstrated cis-eQTL effects on mRNA expression in the temporal cortex and hippocampus. rs2236944(*GNAI2*) was associated with differential expression in frontal cortex and cerebellum. rs16971186(*VPS18*) altered gene expression in whole blood, caudate, and anterior cingulate cortex, suggesting potential peripheral biomarkers of cognitive function.

### 3.4. Bioinformatic Analysis

For pathway analyses, we used a gene-based aggregation approach, in which SNP-level association results of 116 SNPs (Supplementary Table S3) were mapped to genes (based on physical position), and gene-level significance was derived using all SNPs within each gene.

GO Biological Process enrichment identified significant enrichment of the ERBB4 signaling pathway (GO:0038130; adjusted *p* = 5.24 × 10^−3^), driven by the genes *ERBB4*, *NRG1*, and *NRG3.* The enrichment demonstrated a high fold enrichment (91.26), indicating substantial over-representation relative to background expectation.

**Figure 3:**
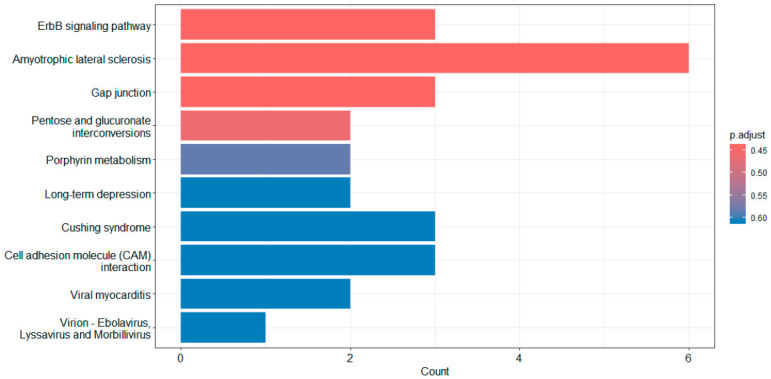
Top 10 KEGG pathways using barplot function. Bar lengths represent the number of genes contributing to each pathway, and color indicates adjusted *p*-values (Benjamini–Hochberg correction).

KEGG pathway enrichment analysis was performed using genes mapped from SNP-level association results that met a significance threshold. The top 20 enriched pathways are listed in Supplementary Table S5 and the top 10 pathways are shown in Figure 3. Among these, the ErbB signaling pathway demonstrated the strongest enrichment signal (*p* = 9.04 × 10^−3^), followed by pathways related to neurodegeneration (amyotrophic lateral sclerosis, *p* = 9.68 × 10^−3^), cellular communication (gap junction, *p* = 9.92 × 10^−3^), and metabolic processes (pentose and glucuronate interconversions, *p* = 1.39 × 10^−2^).

**Figure 4:**
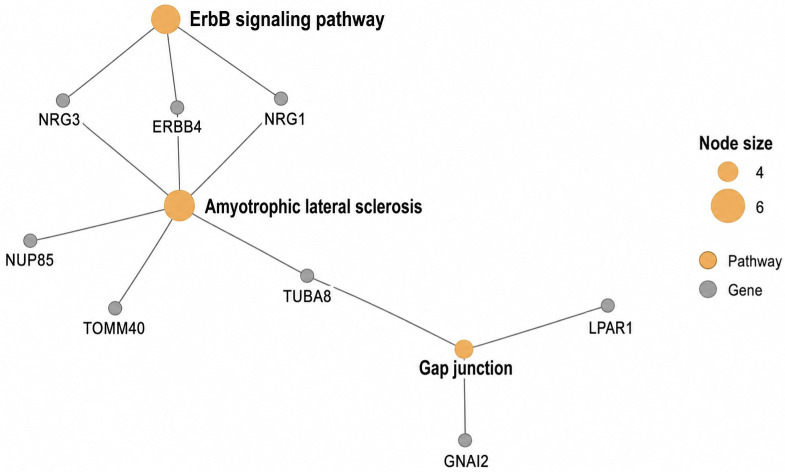
Plot of top 3 KEGG pathways using cnetplot function. Nodes represent genes (gray) and enriched pathways (orange), with edges indicating gene–pathway relationships.

Network analysis of the top three KEGG pathways (Figure 4) revealed a loosely connected gene–pathway network centered on the Amyotrophic lateral sclerosis pathway, which acts as a hub linking multiple genes, including *ERBB4*, *NRG1*, *NRG3*, *TUBA8*, *TOMM40*, and *NUP85*. The *ErbB* signaling pathway is directly connected to this hub through shared genes (*ERBB4*, *NRG1*, *NRG3*), suggesting the involvement of receptor-mediated signaling. A secondary branch involving the Gap junction pathway connects genes such as *GNAI2*, *LPAR1*, and *TUBA8*, indicating potential roles in cell–cell communication.

**Figure 5:**
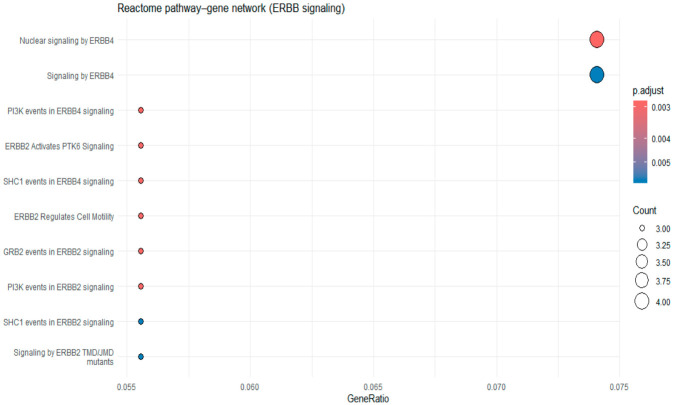
Top 10 Reactome pathway analysis using dotplot function. The x-axis represents gene ratio, dot size indicates the number of genes, and color reflects adjusted *p* values.

We further conducted a focused pathway analysis using Reactome gene sets. The top 20 Reactome pathways are listed in Supplementary Table S6 and the top 10 pathways are shown in Figure 5. As shown in Figure 5, multiple ERBB-related subpathways were enriched, including signaling by ERBB4, nuclear signaling by ERBB4, and downstream signaling modules such as PI3K events in ERBB4 signaling and SHC1-mediated signaling.

**Figure 6:**
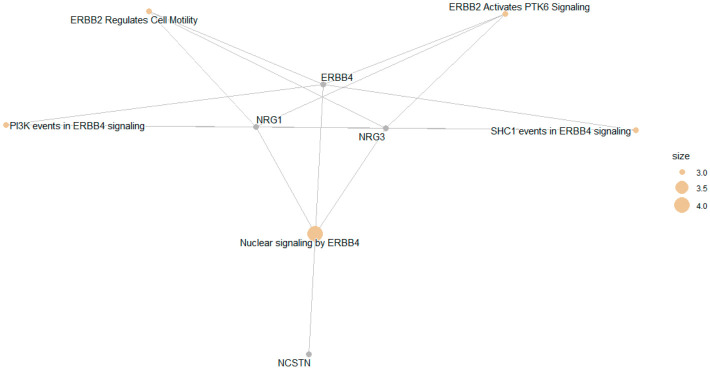
Plot of top 5 Reactome pathways using cnetplot function. Nodes represent genes (gray) and enriched Reactome pathways (orange), with edges indicating gene membership within pathways. Node size reflects the number of contributing genes per pathway.

To consider the potentially biological complexity in which a gene may belong to multiple annotation categories and to provide information on numeric changes if available, we used the cnetplot function to extract the complex association. A gene–pathway network analysis of top five pathways revealed that multiple enriched Reactome pathways were interconnected through *ERBB4*, *NRG1*, and *NRG3,* highlighting *ERBB4*-mediated signaling as the central biological axis underlying the enrichment results (Figure 6). Reactome pathway enrichment analysis identified significant overrepresentation of ERBB4 signaling–related pathways, including PI3K events in ERBB4 signaling (adjusted *p* = 2.82 × 10^−3^, FDR < 0.01). Key driver genes included *ERBB4*, *NRG1*, and *NRG3,* suggesting activation of neuregulin-mediated receptor tyrosine kinase signaling (Figure 6 and Supplementary Table S6). In particular, ERBB4 serves as a central hub linking multiple signaling processes, while NRG1 and NRG3 contribute to upstream ligand-mediated activation of ERBB receptors. Additional genes such as NCSTN appear in more limited connections within the network.

#### SNPs in ERBB4 Signaling Pathway

As shown in Supplementary Table S3, rs1521543 in *ERBB4* was significantly associated with Digit Span Backward (*p* = 1.66 × 10^−8^) and showed additional associations with Logical Memory IIA (*p* = 5.91 × 10^−5^), Digit Ordering (*p* = 7.34 × 10^−5^), and other cognitive measures. Similarly, rs16875655 in *NRG1* was significantly associated with Digit Span Forward (*p* = 1.75 × 10^−8^) and also demonstrated associations with Digit Span Backward (*p* = 6.14 × 10^−7^), Logical Memory IIA (*p* = 6.87 × 10^−4^), and Digit Ordering (*p* = 6.21 × 10^−4^). In addition, rs1937957 in *NRG3* was significantly associated with Animal Fluency (*p* = 4.65 × 10^−8^) and showed broader associations across multiple domains, including Logical Memory IA (*p* = 3.29 × 10^−5^), Logical Memory IIA (*p* = 2.64 × 10^−5^), Digit Span Forward (*p* = 2.05 × 10^−4^), Digit Span Backward (*p* = 3.15 × 10^−6^), and Digit Ordering (*p* = 9.80 × 10^−7^). These results showed shared genetic variants among these cognitive measures.

## 4. Discussion

This family-based GWAS of eight cognitive phenotypes in 3007 individuals from the NIA-LOAD cohort identified 116 genome-wide significant variants associated with episodic memory, working memory, and semantic fluency. The strongest signal was observed at rs2075650 within the TOMM40/APOE region, consistent with prior AD genetics. Additional loci implicated genes involved in synaptic function and neurodevelopment, including PTPRD, NCAM2, GRM7, and NRG3. Notably, Digit Span Backward yielded the largest number of associations, highlighting the potential sensitivity of working memory measures to genetic variation. Cross-phenotype overlap was observed across multiple domains, suggesting shared genetic influences. Functional annotation indicated regulatory potential for several variants, including transcription factor binding and splicing effects. Pathway analyses consistently implicated ERBB4-related signaling, including neuregulin-mediated PI3K pathways. These findings support a polygenic architecture in which both shared and domain-specific genetic influences contribute to cognitive variability [17,51].

### 4.1. Cognitive Genetics in Alzheimer’s Disease

Cognitive decline in AD reflects progressive synaptic dysfunction and neurodegeneration that begin years before clinical diagnosis [6,52]. While the *APOE* ε4 allele remains the strongest genetic risk factor for late-onset AD, it explains only a portion of the variability in cognitive trajectories [14]. Increasing evidence from large-scale GWAS indicates that cognitive performance is influenced by many variants of small effect, implicating pathways related to synaptic structure, neuronal development, and plasticity [17,53]. Most prior studies have focused on global cognitive ability or case–control disease status [54]. In contrast, using domain-specific quantitative phenotypes in the present study allows for a more precise characterization of genetic effects on distinct cognitive systems. Episodic memory, working memory, and semantic fluency exhibit differential vulnerability across the AD continuum, and genetic influences may vary accordingly. By leveraging a family-based design, this study reduces confounding due to population stratification and may enhance detection of heritable effects, although generalizability remains a consideration.

### 4.2. Key Loci and Biological Interpretation

The strongest association identified in this study, rs2075650 within the TOMM40/APOE region, likely reflects APOE-related effects rather than an independent TOMM40 signal, given the well-established linkage disequilibrium between these loci. This variant, previously associated with AD risk [13,14], also influenced recall ability, suggesting shared pathways between memory encoding and AD pathology. Variants in *TOMM40* have been linked to both AD risk and age of onset independent of *APOE* [55,56,57]. The present study furthermore added that the *TOMM40* (rs2075650) was significantly associated with AD as a binary outcome (*p* = 4.60 × 10^−24^) (Table 3).

Beyond the TOMM40/APOE region, several loci implicated genes involved in synaptic structure and neuronal connectivity. Amyloid precursor-like protein 2 (*APLP2*) was associated with delayed recall. *APLP2* shares structural homology with *APP*, the gene encoding amyloid-β precursor protein, and plays roles in synapse formation and neuronal migration [58]. While *APLP2* itself does not produce amyloid-β, it interacts with *APP* to regulate synaptic remodeling [59]. Similarly, *NCAM2* (Neural Cell Adhesion Molecule 2) was also associated with delayed recall. *NCAM2* facilitates synapse maintenance and axonal targeting, and its loss has been observed in early AD stages [60,61]. *NCAM2* disruption leads to dendritic spine loss and impaired long-term potentiation (LTP), key substrates of memory formation. This association supports the notion that cognitive decline in AD results not only from neurodegeneration but also from genetic susceptibility to synaptic disassembly.

PTPRD emerged as a recurrent locus across multiple cognitive domains. This gene encodes a receptor-type protein tyrosine phosphatase involved in synaptic organization and axon guidance. Beyond neurodevelopmental roles, common variants in *PTPRD* have been associated with attention-deficit/hyperactivity disorder, cognitive performance, addiction vulnerability, and sleep regulation, suggesting pleiotropic effects on higher-order cognitive processes [62]. Importantly, emerging neuropathological evidence links *PTPRD* to AD-related pathogenic processes. Transcriptomic and postmortem analyses indicate that *PTPRD* expression correlates with tau pathology and synaptic vulnerability in AD brains [63]. Given that tau-mediated synaptic dysfunction precedes overt neuronal loss [52], variation in *PTPRD* may influence individual susceptibility to synaptic destabilization under neurodegenerative stress. Our research findings extend prior work by implicating *PTPRD* specifically in working memory and executive performance, potentially through modulation of synaptic plasticity and long-range cortical connectivity.

Working memory phenotypes highlighted additional loci, including *GRM7*, *DNER*, and *PAX5*. Novel associations with Digit Span and Digit Ordering tests involved *GRM7* (glutamate receptor metabotropic 7), *DNER* (Delta/Notch-like EGF-related receptor), and *PAX5* (Paired box 5). *GRM7* modulates glutamatergic neurotransmission and has been associated with cognitive performance and neuropsychiatric traits [64,65]. *DNER* is involved in Notch signaling and neuronal differentiation [66], while *PAX5* regulates neural lineage commitment during development [67,68]. These genes highlight the contribution of excitatory signaling and developmental transcriptional programs to working memory integrity in aging. For Digit Span Backward, 90 genome-wide significant loci were identified, with the top seven signals being rs11894104 (*HSPC159||AFTPH*), rs2236944 (*GNAI2*), rs410513 (*LOC100129568||LOC137012*), rs4312758 (*KIAA0232*), rs6880825 (*YTHDC2||KCNN2*), rs7148156 (*GOLGA5*), and rs7571817 (*DNER*). *GNAI2* plays a role in neurodevelopmental signaling [69]. For Digit Ordering, six genome-wide significant loci were detected: rs16971186 (*VPS18*), rs6714929 (*ICOS||LOC100132132*), rs4878454 (*MOBKL2B*), rs2988003 (*PAX5*), rs2381595 (*PAX5*), and rs9617659 (*PEX26||TUBA8*). *PAX5* regulates neural lineage commitment during development [67,68]. These genes highlight the contribution of excitatory signaling and developmental transcriptional programs to working memory integrity in aging.

A single SNP, rs1937957 (*NRG3)*, reached genome-wide significance for Animal Fluency (*p* = 4.7 × 10^−8^). *NRG3* encodes neuregulin-3, a ligand in the ErbB4 signaling pathway that regulates cortical circuit maturation and cognitive flexibility [70,71]. Prior work has linked *NRG3* to schizophrenia, impulsivity, and cognitive adaptability. Its role in semantic fluency, an executive-linguistic task, suggests shared molecular underpinnings between psychiatric and neurodegenerative cognitive phenotypes.

### 4.3. Cross-Phenotype Overlap

We observed the overlap of loci across multiple cognitive domains, particularly for PTPRD and the APOE/TOMM40 region. Such overlap suggests potential shared genetic influences across memory and executive function. *PTPRD* emerged as a recurrent locus across Logical Memory II, Digit Span Forward, and Digit Ordering, indicating potential pleiotropic effects spanning episodic memory and executive control processes. *PTPRD* encodes a receptor-type protein tyrosine phosphatase implicated in synaptic organization and axon guidance, with emerging evidence supporting its role in neuronal connectivity and cognitive performance [72]. Similarly, *TOMM40* variants influenced both episodic memory and semantic fluency, consistent with prior findings linking this locus to cognitive resilience and metabolic integrity in aging populations [56,57,73]. *TOMM40* encodes a key component of the mitochondrial translocase complex and is in strong linkage disequilibrium with *APOE*, a well-established AD risk gene [55]. Collectively, these findings highlight shared molecular mechanisms influencing multiple cognitive systems and underscore the importance of synaptic and mitochondrial pathways in late-life cognitive function. The SNP overlap is consistent with the brain and behavior overlap of episodic memory and working memory, indicating shared polygenic influences on distinct but related cognitive systems [17,74]. However, we did not perform formal tests of pleiotropy or genetic correlation; therefore, these findings should be interpreted as suggestive rather than definitive evidence of shared genetic architecture.

### 4.4. Integrative Perspective: Shared and Domain-Specific Genetic Architecture

Together, our findings suggest a combination of shared and domain-specific genetic influences on cognition in AD. Overlapping loci, such as those in PTPRD and the APOE region, point to common pathways influencing multiple cognitive domains, while domain-specific signals, including NRG3 and PAX5, indicate distinct genetic contributions. This pattern aligns with recent multivariate GWAS demonstrating extensive genetic correlations among cognitive abilities [17,74]. Moreover, domain-specific loci such as *NRG3* and *PAX5* emphasize that distinct neural networks may also have unique genetic determinants. Our pathway enrichment analysis reinforces the convergence of associated genes on synaptic transmission, neuron projection, and calcium signaling, consistent with neurobiological models of AD that emphasize synaptic dysfunction as a primary driver of cognitive impairment [52,75]. The enrichment of glutamatergic synapse and axon guidance pathways further supports the involvement of excitatory signaling and connectivity in sustaining cognitive function.

### 4.5. Comparison with Previous GWAS

Several loci identified in this study overlap with findings from previous GWAS of AD and cognitive function. *TOMM40* and *PTPRD* have repeatedly emerged as AD risk modifiers [13,14], while *GRM7* and *GNAI2* have been linked to intelligence and working memory in population cohorts [17,51]. The replication of these signals in a family-based AD cohort supports their robustness and relevance across cognitive phenotypes. In contrast, the identification of *NRG3*, *DNER*, and *PAX5* provides novel leads for future research. These genes are less frequently studied in AD but play essential roles in neurodevelopment and synaptic maintenance, suggesting they may influence cognitive resilience or vulnerability. However, given the modest sample size relative to large population GWAS, replication in independent datasets is essential.

### 4.6. Pathway Enrichment

Pathway analyses consistently identified enrichment of ERBB4-related signaling pathways, driven by ERBB4, NRG1, and NRG3. Neuregulin–ERBB4 signaling plays a critical role in synaptic plasticity, interneuron development, and maintenance of excitatory–inhibitory balance in the cortex [76]. *ERBB4* is highly expressed in GABAergic interneurons and modulates NMDA receptor function and synaptic transmission, processes fundamental to learning and memory [77,78]. Importantly, *PI3K* signaling downstream of ERBB receptors regulates neuronal survival and resilience against amyloid-β-induced toxicity, suggesting a plausible mechanistic link to LOAD pathophysiology [79,80]. However, these results were based on a limited set of genes and included loci with varying levels of statistical support. Therefore, pathway findings should be interpreted as exploratory and hypothesis-generating rather than definitive evidence of pathway-level involvement.

### 4.7. Strengths and Limitations

This study has several strengths. The family-based design reduces confounding due to population stratification and allows detection of genetic effects within pedigrees. The use of quantitative cognitive phenotypes enhances sensitivity to genetic influences compared to case–control designs. Additionally, the analysis of multiple cognitive domains provides a comprehensive view of cognitive genetics in AD. However, limitations should be noted. Sample sizes for some phenotypes were modest, limiting power to detect small-effect variants. The use of the QFAM-total test increases power but may be sensitive to residual population structure. APOE genotypes were not explicitly modeled, and associations in the TOMM40 region likely reflect APOE-related effects. Furthermore, pathway analyses were based on suggestive and aggregated signals, and functional annotation does not establish causality. Finally, replication in independent cohorts is required to confirm these findings.

### 4.8. Implications and Future Directions

These findings contribute to understanding the genetic architecture of cognitive function in AD and highlight potential targets for future investigation. Integrating cognitive GWAS with functional genomics, transcriptomics, and longitudinal data may clarify mechanisms linking genetic variation to cognitive decline. Future studies should incorporate APOE-adjusted analyses, larger sample sizes, and diverse populations. Additionally, polygenic approaches may improve prediction of cognitive trajectories and disease progression.

## 5. Conclusions

In summary, this family-based GWAS identified multiple genetic loci associated with domain-specific cognitive phenotypes in AD. The results support a polygenic architecture involving genes related to synaptic function, neuronal signaling, and development. While several findings are consistent with prior AD genetics, others represent novel associations requiring replication. Pathway analyses suggest the involvement of ERBB-related signaling, although these findings remain exploratory. Overall, this study highlights the value of quantitative cognitive traits in uncovering genetic influences on cognition and provides a foundation for future research aimed at understanding and mitigating cognitive decline in AD.

## Figures and Tables

**Table 1:** Demographic variables.

Variable	N/Mean	Non-AD (N, %)/(Mean, SD)	AD (N, %)/(Mean, SD)	χ^2^/*t*	*p*
Sex					
Male	901	466, 51.7	435, 48.3	1.20	0.2739
Female	1644	813, 49.5	831, 50.5		
Age (year)	76.2	75.5, 8.14	77.3, 10.0	−0.44	0.6767
Race					
White	2270	1061, 46.7	1209, 53.3	26.75	<0.0001
African American	114	77, 6.5	37, 32.5		
Other	18	2, 12.5	14, 87.5		
Education (year)	14.0	14.7, 2.9	12.8, 3.3	12.66	<0.0001

AD = Alzheimer’s disease; SD = Standard deviation.

**Table 2:** Descriptive characteristics of 8 cognitive functions.

Variable	N	Non-AD (Mean, SD)	AD (Mean, SD)	*t*, *p*
Logical Memory IA—Immediate Recall	1411	13.1, 4.0	2.9, 3.6	37.99, <0.0001
Logical Memory IIA—Delayed Recall	1400	12.0, 4.2	1.7, 2.8	44.66, <0.0001
Digit Span Forward	1410	9.1, 2.0	6.3, 3.2	13.07, <0.0001
Digit Span Backward	1404	6.8, 2.3	3.6, 2.5	17.32, <0.0001
Digit Ordering	984	7.8, 1.8	3.6, 2.9	19.57, <0.0001
Animal Fluency	1505	19.5, 5.5	7.9, 5.4	29.90, <0.0001
Fruit and Vegetable Fluency	460	17.9, 5.1	6.9, 5.1	19.03, <0.0001
Vegetable Fluency	954	14.7, 4.1	5.8, 4.2	21.63, <0.0001

AD = Alzheimer’s disease; SD = Standard deviation.

**Table 3:** Top SNPs associated with delayed recalls, immediate recall and animal fluency (*p* < 5 × 10^−8^).

Chr.	SNP	Position ^a^	Nearest Gene ^b^	MA ^c^	MAF ^d^	*p*-AD ^e^	*p*-LM IIA ^f^	*p*-LM IA ^g^	*p_*Animal ^h^	*p*-DigBak ^i^	*p*-DigFor ^j^	*p*-DigOrd ^k^
19	rs2075650	50087459	*TOMM40*	G	0.14	4.60 × 10^−24^ *	1.23 × 10^−10^ *	5.66 × 10^−9^ *	5.64 × 10^−5^	2.84 × 10^−6^	5.57 × 10^−7^	2.7 × 10^−4^
11	rs12281267	129492310	*APLP2*	A	0.13	8.47 × 10^−4^	5.50 × 10^−9^ *	4.78 × 10^−6^	1.82 × 10^−4^	3.71 × 10^−6^	5.58 × 10^−6^	3.81 × 10^−7^
9	rs1353983	8308948	*PTPRD*	A	0.42	6.84 × 10^−3^	8.87 × 10^−9^ *	1.48 × 10^−6^	1.92 × 10^−3^	5.81 × 10^−3^	1.78 × 10^−5^	1.76 × 10^−4^
9	rs970347	8289968	*C9orf123||PTPRD*	A	0.29	7.67 × 10^−3^	1.10 × 10^−8^	5.07 × 10^−6^	1.28 × 10^−3^	1.29 × 10^−2^	1.19 × 10^−3^	5.52 × 10^−4^
9	rs1392511	8287668	*C9orf123||PTPRD*	A	0.28	4.68 × 10^−3^	1.42 × 10^−8^	1.33 × 10^−6^	6.44 × 10^−4^	2.61 × 10^−2^	1.67 × 10^−3^	8.95 × 10^−5^
11	rs959354	129514791	*APLP2*	G	0.15	8.47 × 10^−5^	2.60 × 10^−8^	4.13 × 10^−6^	2.29 × 10^−4^	1.45 × 10^−4^	2.05 × 10^−5^	4.96 × 10^−6^
21	rs2826728	21484517	*NCAM2*	G	0.07	0.9927	2.66 × 10^−8^	4.59 × 10^−7^	2.69 × 10^−5^	6.89 × 10^−9^ *	6.64 × 10^−7^	7.11 × 10^−5^
10	rs1937957	84113905	*NRG3*	G	0.26	0.4803	2.64 × 10^−5^	3.29 × 10^−5^	4.65 × 10^−8^	3.15 × 10^−6^	2.05 × 10^−4^	9.80 × 10^−7^

^a^ Physical position is based on NCBI36 (hg18); ^b^ SNP function; ^c^ Minor allele; ^d^ MAF refers to the minor allele frequency; ^e^ *p*-value for AD; ^f^ *p*-value for Logical Memory IIA (Delayed Recall); ^g^ *p*-value for Logical Memory IA (Immediate Recall); ^h^ *p*-value for Animal Fluency; ^i^ *p*-value for Digit Span Backward; ^j^ *p*-value for Digit Span Forward; ^k^ *p*-value for Digit Ordering. * *p* < 1.04 × 10^−8^.

**Table 4:** Top SNPs associated with digital span phenotypes (*p* < 5 × 10^−8^).

Chr.	SNP	Position ^a^	Nearest Gene ^b^	MA ^c^	MAF ^d^	*p*-AD ^e^	*p*-LM IIA ^f^	*p*-LM IA ^g^	*p_*Animal ^h^	*p*-DigBa ^i^	*p*-DigFor ^j^	*p*-DigOrd ^k^
2	rs11894104	64567515	*HSPC159||AFTPH*	G	0.01	0.9662	1.28 × 10^−5^	3.70 × 10^−5^	7.12 × 10^−8^	6.51 × 10^−12^ *	8.94 × 10^−7^	4.45 × 10^−7^
4	rs4312758	6925418	*KIAA0232*	A	0.11	0.0513	6.95 × 10^−7^	1.83 × 10^−6^	5.35 × 10^−8^	1.33 × 10^−10^ *	8.76 × 10^−9^ *	3.49 × 10^−6^
14	rs7148156	92334286	*GOLGA5*	G	0.03	0.1302	2.25 × 10^−4^	2.50 × 10^−3^	1.22 × 10^−3^	1.85 × 10^−10^ *	2.63 × 10^−6^	1.02 × 10^−3^
5	rs6880825	113382613	*YTHDC2||KCNN2*	G	0.05	0.1734	1.14 × 10^−4^	2.66 × 10^−4^	1.73 × 10^−4^	2.66 × 10^−10^ *	3.89 × 10^−7^	9.91 × 10^−5^
3	rs2236944	50267197	*GNAI2*	T	0.07	0.7075	8.93 × 10^−8^	9.54 × 10^−5^	2.65 × 10^−6^	7.02 × 10^−10^ *	3.08 × 10^−7^	5.95 × 10^−8^
2	rs7571817	230000219	*DNER*	A	0.02	0.3831	1.53 × 10^−5^	2.85 × 10^−4^	2.07 × 10^−4^	1.78 × 10^−9^ *	3.32 × 10^−7^	2.63 × 10^−4^
8	rs410513	15693905	*LOC100129568||LOC137012*	G	0.02	0.6391	1.08 × 10^−6^	1.45 × 10^−5^	1.24 × 10^−6^	1.83 × 10^−9^ *	7.67 × 10^−6^	3.84 × 10^−7^
9	rs10759013	8865954	*PTPRD*	A	0.13	3.00 × 10^−3^	1.07 × 10^−2^	1.03 × 10^−1^	6.97 × 10^−2^	2.00 × 10^−7^	7.20 × 10^−10^ *	3.56 × 10^−4^
3	rs6788201	7101102	*GRM7*	C	0.03	0.9976	6.48 × 10^−6^	4.24 × 10^−4^	7.93 × 10^−3^	1.09 × 10^−7^	8.53 × 10^−10^ *	2.19 × 10^−4^
1	rs3862188	245932396	*OR13G1||OR6F1*	C	0.28	0.4689	7.56 × 10^−4^	3.25 × 10^−3^	7.99 × 10^−4^	2.67 × 10^−9^ *	4.84 × 10^−9^ *	5.84 × 10^−4^
1	rs880143	245934736	*OR13G1||OR6F1*	G	0.28	0.4689	8.10 × 10^−4^	3.23 × 10^−3^	7.78 × 10^−4^	3.20 × 10^−9^ *	5.95 × 10^−9^ *	5.83 × 10^−4^
14	rs11160011	91063617	*SMEK1||PP8961*	A	0.03	0.8511	1.15 × 10^−3^	1.54 × 10^−3^	6.48 × 10^−3^	1.06 × 10^−8^	8.18 × 10^−9^ *	7.04 × 10^−6^
10	rs1110399	72770272	*SLC29A3*	C	0.13	0.4300	2.17 × 10^−3^	3.50 × 10^−4^	4.77 × 10^−3^	1.26 × 10^−6^	8.56 × 10^−9^ *	3.39 × 10^−3^
15	rs16971186	38977347	*VPS18*	C	0.02	2.82 × 10^−2^	2.60 × 10^−2^	3.29 × 10^−2^	3.05 × 10^−1^	9.21 × 10^−2^	2.15 × 10^−1^	1.67 × 10^−8^
2	rs6714929	204770878	*ICOS||LOC100132132*	A	0.17	0.1327	4.39 × 10^−6^	5.48 × 10^−6^	3.21 × 10^−4^	9.92 × 10^−6^	2.01 × 10^−5^	3.24 × 10^−8^
9	rs4878454	27426033	*MOBKL2B*	A	0.01	3.20 × 10^−3^	7.86 × 10^−7^	2.24 × 10^−5^	2.59 × 10^−4^	1.20 × 10^−5^	2.88 × 10^−4^	4.21 × 10^−8^
9	rs2988003	37008949	*PAX5*	G	0.03	0.3041	2.35 × 10^−5^	5.29 × 10^−4^	2.30 × 10^−3^	5.20 × 10^−6^	5.33 × 10^−4^	4.21 × 10^−8^
9	rs2381595	37007666	*PAX5*	A	0.03	0.2898	1.04 × 10^−4^	1.64 × 10^−3^	2.67 × 10^−3^	6.01 × 10^−6^	4.25 × 10^−4^	4.29 × 10^−8^
22	rs9617659	16966756	*PEX26||TUBA8*	G	0.01	0.9002	9.30 × 10^−5^	9.56 × 10^−5^	7.11 × 10^−3^	1.45 × 10^−7^	4.84 × 10^−5^	4.97 × 10^−8^

^a^ Physical position is based on NCBI36 (hg18); ^b^ SNP function; ^c^ Minor allele; ^d^ MAF refers to the minor allele frequency; ^e^ *p*-value for AD; ^f^ *p*-value for Logical Memory IIA (Delayed Recall); ^g^ *p*-value for Logical Memory IA (Immediate Recall); ^h^ *p*-value for Animal Fluency; ^i^ *p*-value for Digit Span Backward; ^j^ *p*-value for Digit Span Forward; ^k^ *p*-value for Digit Ordering. * *p* < 1.04 × 10^−8^.

**Table 5:** Five flanking SNPs within TOMM40 and/or APOE.

Chr.	SNP	Position ^a^	Nearest Gene ^b^	MA ^c^	MAF ^d^	*p*-AD ^e^	*p*-LM IIA ^f^	*p*-LM IA ^g^	*p_*Animal ^h^	*p*-DigBak ^i^	*p*-DigFor ^j^	*p*-DigOrd ^k^
19	rs157580	50087106	*TOMM40*	G	0.374	8.89 × 10^−8^	3.72 × 10^−7^	1.42 × 10^−5^	0.008936	0.05556	0.000484	0.03321
19	rs2075650	50087459	*TOMM40*	G	0.14	4.60 × 10^−24^ *	1.23 × 10^−10^ *	5.66 × 10^−9^ *	5.64 × 10^−5^	2.84 × 10^−6^	5.57 × 10^−7^	2.37 × 10^−4^
19	rs8106922	50093506	*TOMM40*	G	0.37	6.49 × 10^−8^	0.01811	0.02826	0.01814	6.09 × 10^−6^	0.002185	0.001193
19	rs405509	50100676	*APOE*	A	0.47	3.70 × 10^−7^	0.07093	0.09685	0.03195	0.002144	0.09525	0.08696
19	rs769451	50102751	*APOE*	G	0.01	0.3955	0.8145	0.8575	0.928	0.1116	0.1528	0.6203

^a^ Physical position is based on NCBI36 (hg18); ^b^ SNP function; ^c^ Minor allele; ^d^ MAF refers to the minor allele frequency; ^e^ *p*-value for AD; ^f^ *p*-value for Logical Memory IIA (Delayed Recall); ^g^ *p*-value for Logical Memory IA (Immediate Recall); ^h^ *p*-value for Animal Fluency; ^i^ *p*-value for Digit Span Backward; ^j^ *p*-value for Digit Span Forward; ^k^ *p*-value for Digit Ordering. * *p* < 1.04 × 10^−8^.

## Data Availability

Data used in this study were obtained from the National Institute on Aging Late-Onset Alzheimer’s Disease Family Study (NIA-LOAD) via the Database of Genotypes and Phenotypes (study accession: phs000168.v2.p2). These data are available to qualified researchers upon application and approval through dbGaP (https://dbgap.ncbi.nlm.nih.gov/beta/study/phs000168.v2.p2/#study) (accessed on 31 August 2025).

## Supplementary Materials

The following supporting information can be downloaded at https://www.mdpi.com/article/10.3390/cimb48050442/s1.

## Abbreviations

The following abbreviations are used in this manuscript:

AD	Alzheimer’s disease
eQTL	Expression quantitative trait loci
ESEs	Exonic splicing enhancers
ESSs	Exonic splicing silencers
GO	Gene Ontology
GWAS	Genome-wide association studies
HWE	Hardy–Weinberg equilibrium
KEGG	Kyoto Encyclopedia of Genes and Genomes
LM IA	Logical Memory IA (Immediate Recall)
LM IIA	Logical Memory IIA (Delayed Recall)
LOAD	Late-Onset Alzheimer’s Disease
MAF	Minor allele frequency
MCI	Mild cognitive impairment
QC	Quality control
SNP	Single nucleotide polymorphisms
SD	Standard deviation
TFBS	Transcription factor binding site
